# Systematic review and meta-analysis of ivermectin for treatment of COVID-19: evidence beyond the hype

**DOI:** 10.1186/s12879-022-07589-8

**Published:** 2022-07-23

**Authors:** Milena Soriano Marcolino, Karina Cardoso Meira, Nathalia Sernizon Guimarães, Paula Perdigão Motta, Victor Schulthais Chagas, Silvana Márcia Bruschi Kelles, Laura Caetano de Sá, Reginaldo Aparecido Valacio, Patrícia Klarmann Ziegelmann

**Affiliations:** 1 Department of Internal Medicine, Medical School and Telehealth Center, University Hospital, Universidade Federal de Minas Gerais, Avenida Professor Alfredo Balena 190, sala 246, Belo Horizonte, 30130-100 Brazil; 2 Institute for Health Technology Assessment (IATS/CNPq), Rua Ramiro Barcelos, 2359, Prédio 21|Sala 507, Porto Alegre, Brazil; 3 Health School, Federal University of Rio Grande do Norte, Av. Sen. Salgado Filho, s/n-Lagoa Nova, Natal, Rio Grande do Norte, Brazil; 4 Instituto de Saúde Coletiva da Universidade Federal da Bahia, R. Basílio da Gama, s/n-Canela, Salvador, Brazil; 5 Faculdade de Farmácia da Universidade Federal de Minas Gerais, R. Prof. Moacir Gomes de Freitas S/N-Pampulha, Belo Horizonte, Minas Gerais Brazil; 6 Department of Medicine and Nursing, Universidade Federal de Viçosa, Av. Peter Henry Rolfs, University Campus, Viçosa, Brazil; 7 Pontifícia Universidade Católica de Minas Gerais, R. do Rosário, 1.081 Bairro Angola, Betim, Brazil; 8 Unimed-BH, Belo Horizonte, MG Brazil; 9 Faculdade Ciências Médicas de Minas Gerais-FCMMG, Alameda Ezequiel Dias, Belo Horizonte, 275 Brazil; 10 Hospital Metropolitano Odilon Behrens, R. Formiga, 50, Belo Horizonte, Brazil; 11 Epidemiology e Statistics Department, Universidade Federal do Rio Grande do Sul, Rua Ramiro Barcelos, Porto Alegre, RS 2400 Brazil

**Keywords:** COVID-19, SARS-CoV-2, Novel coronavirus, Therapeutics, Systematic review, Meta-analysis, Ivermectin, Evidence-based medicine, Mortality

## Abstract

**Background:**

The role of ivermectin in the treatment of COVID-19 is still under debate, yet the drug has been widely used in some parts of the world, as shown by impressive market data. The available body of evidence may have changed over the last months, as studies have been retracted and “standards of care” (SOC) used in control groups have changed with rapidly evolving knowledge on COVID-19. This review aims to summarize and critically appraise the evidence of randomized controlled trials (RCTs) of ivermectin, assessing clinical outcomes in COVID-19 patients.

**Methods:**

RCTs evaluating the effects of ivermectin in adult patients with COVID-19 were searched through June 22, 2022, in four databases, L.OVE platform, clinical trial registries and pre-prints platforms. Primary endpoints included all-cause mortality and invasive ventilation requirement. Secondary endpoint was the occurrence of adverse events. Risk of bias was evaluated using the Cochrane Risk of Bias 2.0 tool. Meta-analysis included only studies which compared ivermectin to placebo or SOC. Random-effects were used to pool the risk ratios (RRs) of individual trials. The quality of evidence was evaluated using GRADE. The protocol was register in PROSPERO (CRD42021257471).

**Results:**

Twenty-five RCTs fulfilled inclusion criteria (n = 6310). Of those, 14 compared ivermectin with placebo, in night ivermectin associated with SOC was compared to SOC and two studies compared ivermectin to an active comparator. Most RCTs had some concerns or high risk of bias, mostly due to lack of concealment of the randomization sequence and allocation, lack of blinding and high number of missing cases. Ivermectin did not show an effect in reducing mortality (RR = 0.76; 95%CI: 0.52–1.11) or mechanical ventilation (RR = 0.74; 95%CI: 0.48–1.16). This effect was consistent when comparing ivermectin vs. placebo, and ivermectin associated with SOC vs. SOC, as well as in sensitivity analysis. Additionally, there was very low quality of evidence regarding adverse effects (RR = 1.07; 95%CI: 0.84–1.35).

**Conclusions:**

The evidence suggests that ivermectin does not reduce mortality risk and the risk of mechanical ventilation requirement. Although we did not observe an increase in the risk of adverse effects, the evidence is very uncertain regarding this endpoint.

**Supplementary Information:**

The online version contains supplementary material available at 10.1186/s12879-022-07589-8.

## Background

Despite the efforts and the relative success of vaccination against the coronavirus disease 2019 (COVID-19) worldwide, it is possible that the pandemic will persist for a long period, due to the ascension of outcoming variants and anti-vaccine movements around the world [1–3]. In this context, several drugs, alone or in combination with other drugs, vitamins and minerals were studied to verify the possibility of mitigating the effects of the severe acute respiratory syndrome coronavirus 2 (SARS-CoV-2) or the symptoms of covid-19 [4]. However, until the present date, few pharmacological therapies have been shown to be effective in reducing the number of hospitalizations, mechanical ventilation and death [5, 6].

Using existing medicines that are widely available and at low cost has appeal [7]. The anti-parasite drug ivermectin has grabbed a lot of attention as a potential drug to treat COVID-19, and has become widely used off label, especially in Latin America, to control COVID-19 [8, 9]. The drug has been used for decades to treat parasitic infections, and it is on the list of essential drugs from the World Health Organization (WHO), being considered safe and effective once it is administered in appropriate doses. Several in vitro studies have shown an antiviral effect of ivermectin for flaviviruses, such as Dengue, yellow fever, Zika virus and SARS-CoV-2 as well [10–12]. In cells infected by SARS-CoV-2, ivermectin has been shown to inhibit the attachment of the virus's spike protein to the human cell membrane, which was able to reduce the viral RNA concentration by almost 5000-fold, what increased hopes for clinical benefit in prevention and treatment of COVID-19 [10, 13, 14]. Nonetheless, for the antiviral effect observed in cell culture to be effective in humans, the dose of ivermectin required would be 17 times higher than the maximum safe dosage allowed per day for patients [10].

The enthusiasm brought by the mechanistic effect has also been questioned in a recent network meta-analysis on the effects of ivermectin on viral clearance. When authors analyzed the data using a fixed effects model approach, there was a significant effect of ivermectin on reducing viral clearance (OR 2.32, 95% equal-tailed credible intervals [CrIs] 1.38–3.94); this effect was nonsignificant when they used a random effects model approach (OR 2.70, 95% CrI [1.24, 6.12]), [15] demonstrating how choosing or not the proper methods for analysis can influence the results.

Multiple clinical trials were carried out to assess clinical outcomes [16–19], with conflicting evidence, and some of these studies were withdrawn given concerns about serious data inconsistencies and research fraud [20–24] Yet, the drug has been widely used, and has even been called a “COVID-19 miracle drug”, supported by vaccine opponents, or even by health authorities in some countries [25]. However, there have been reports from different countries of people being hospitalized after self-medicating and developing serious adverse effects due to ivermectin [26]. The need for evidence synthesis is imperative for medical doctors and the community. Systematic reviews have been published on the topic, but in the majority of them a retracted trial represented more than 10% of the overall effect, [20, 21, 27, 28] which overestimated the benefits. One of those was recently reassessed after the exclusion of this trial, which changed the results from reducing mortality to lack of benefit in this outcome [17]. In another systematic review restricted to studies comparing the drug to placebo or standard care, the authors concluded they were uncertain about the efficacy and safety of ivermectin [29].

As the current evidence on the benefits of ivermectin to treat people with COVID-19 is still debatable and there is a risk of serious adverse events, the WHO living guideline recommends that the drug should only be used within clinical trials, and the IDSA's guideline suggests not using it [4, 30]. Therefore, there is still a gap of reliable and updated evidence synthesis of the effect of ivermectin in COVID-19 patients. This study aims to summarize and critically appraise the evidence of randomized controlled trials (RCTs) of ivermectin, assessing clinical outcomes in inpatients or outpatients with COVID-19.

## Methods

This systematic review and meta-analysis was based on recommendations from the Cochrane Guidelines for Systematic Reviews of Interventions and was written according to Preferred Reporting Items for Systematic Reviews and Meta-Analyses (PRISMA) [31, 32]. The review protocol was registered at the PROSPERO (CRD42021257471).

### Search strategy

In order to identify randomized clinical trials assessing the effects of ivermectin use in COVID-19 patients, we searched four independent databases to perform the sensitive literature search: MEDLINE, EMBASE; Central (by Cochrane Library) and Latin American and Caribbean Health Science Information (LILACS). We also searched, on an ongoing basis, the Epistemonikos COVID-19 L·OVE platform (Living Overview of the Evidence), which provides an electronic search in 41 databases, trial registries, preprint servers and other sources (Additional file 7). In the L·OVE platform, we conducted the search by PICO question (patient/population, intervention, comparison/control, outcome). Additionally, we searched for ongoing registered clinical trials at the National Institute of Health United States National Library of Medicine and pre-prints (medRxiv; bioRxiv), and reference lists of included studies and systematic reviews.

There was no language, date, document type, publication status or geographic restriction for inclusion of records. The last search was conducted on March 31, 2022. Descriptors were identified in Medical Subject Headings (MeSH), *Descritores em Ciências da Saúde* (Decs) and Embase Subject Headings (Emtree). The search strategy was adapted based on descriptors in each database and are presented in the Supplementary material. The Cochrane-validated filter for randomized controlled trials was applied [33].

### Outcomes

The primary outcomes were all-cause mortality and invasive ventilation support. Secondary outcome was the percentage of patients who presented adverse events at follow-up.

### Eligibility criteria

We included randomized controlled trials that evaluated patients with confirmed COVID-19, or those with suspicion of COVID-19 by clinical symptoms and imaging findings, which compared systemic ivermectin with placebo, no treatment, standard care (as defined by the researchers in the individual studies) or other drugs for COVID-19 treatment, irrespective of disease severity, and assessed any primary or secondary outcomes. Co‐interventions had to be the same in both study arms. For the assessment of mortality, we included studies which assessed the patients until recovery or death.

Randomized clinical trials which assessed the use of ivermectin in combination with other drugs when compared to placebo were excluded, as we would not be able to assess the effect of ivermectin itself, as well as those which assessed ivermectin for COVID-19 prevention. Studies in which inhaled ivermectin was used as intervention were also excluded.

### Study selection and data extraction

Electronic search results from defined databases were uploaded to the Rayyan Qatar Computing Research Institute [34].

Study selection and data extraction was independently performed by two investigators. A third reviewer resolved any disagreements. For duplicate registrations, only the most recent one was included. Authors initially screened titles and abstracts. Subsequently, they assessed each study to determine whether it met inclusion criteria.

We extracted data on study design (methods, location, setting, inclusion/exclusion criteria, duration and number of participants in each group), participant characteristics (disease severity, age and sex), intervention and comparator characteristics (dose and frequency of ivermectin/comparator, type of comparator, outcome measures at baseline, at the end of follow-up and/or changes in outcome measures from baseline to the end of follow-up).

### Quality assessment

Two investigators independently assessed the risk of bias in the selected studies according to the Cochrane Collaboration’s tool for assessing risk of bias (RoB 2) [35]. Possible sources of bias in randomized trials include random sequence generation, allocation concealment, blinding of participants and personnel, blinding of outcome assessment, incomplete outcome data, selective reporting, and other biases. Three scores of yes, no, and unclear were given to each before mentioned item, referring to high risk, low risk, and unknown risk, respectively**.** We entered and organized our RoB 2 assessments on an Excel spreadsheet (Microsoft Excel RoB2 Macro) [35]. Reviewers resolved discrepancies by discussion.

The overall certainty of the body of evidence was rated by using the Grading of Recommendations Assessment, Development and Evaluation (GRADE) approach, taking into account overall risk of bias, consistency of effect, imprecision, indirectness and publication bias to assess the certainty of the body of evidence [36, 37]. If there were serious concerns in any of these domains, we rated down the quality of evidence. We incorporated the overall RoB2 judgment into our GRADE assessment.

### Statistical analysis

Treatment effects were expressed as risk ratios (RRs), as all outcomes were binary. Pooled RR were calculated using random effects models with the Dersimonian and Laird estimator and the Mantel–Haenszel method, as clinical heterogeneity was expected. Statistical heterogeneity among studies effects were investigated by using Cochran Q test and I2 statistic. Prediction intervals were not used due to the small number of studies in each meta-analysis.

For the main analysis, we opted to summarize the evidence using subgroups: ivermectin vs placebo and ivermectin associated with SOC vs SOC. Different arms of ivermectin were combined in single arms and, in studies with more than two comparator arms, only placebo or SOC was used as the control in the pooled analysis. The trials in which ivermectin was compared to an active drug were presented only in qualitative analysis. As there is evidence of harm with hydroxychloroquine and chloroquine [38, 39], it would not be proper to analyze the comparison of ivermectin and hydroxychloroquine or chloroquine. The second analysis compared ivermectin (associated or not with SOC) to no ivermectin (placebo or SOC).

Sensitivity analyses were pre-specified and performed only for the primary outcomes. The effect size was examined by omitting studies individually, excluding simultaneously studies with extreme results, and also taking into account the risk of bias and the percentage of confirmed individuals of the included studies.

A funnel plot was constructed to assess the possibility of publication bias only for the second analysis due to a small number of studies. The symmetry of the plot was evaluated both visually and formally with Egger’s test. The implications for our results were assessed by the trim-and-fill method. [40, 41].

Analyses were performed in the Rstudio software, version 4.1.0 (R: A Language and Environment for Statistical Computing, Vienna, Austria), by using the ‘Meta’ packages, versions 5.0-0.

### Data availability

The full dataset and statistical codes will be available on reasonable request from any qualified investigator.

## Results

### Search results

**Fig. 1:**
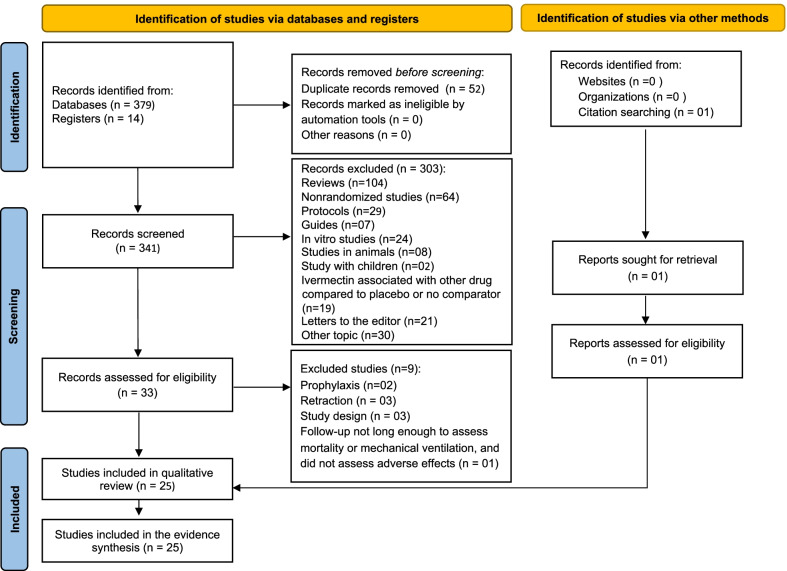
Flow of information through the different phases of the systematic review. The flowchart was adapted from the Preferred Reporting Items for Systematic Review and Meta-Analyses flow chart model

Our search retrieved 379 studies and 14 registers in progress through four selected database searches and the L.OVE platform. After excluding 52 duplicates, 341 titles and abstracts were screened. Full-text articles for the remaining 33 records were retrieved, of which two were excluded for testing the drug for COVID-19 prophylaxis; [42, 43] four for retraction [21–24], two due to the study design (not RCT) [44, 45], one for including children [46], and one for a too short follow-up, having not assessed patients until recovery or death [47] (Fig. 1). One study was found through hand searching reference lists. Therefore, 25 studies were eligible for inclusion in this systematic review. No additional articles were retrieved from the reference lists of the included studies. Of the 25 included studies, 06 were available as preprints upon the time of submission of the present study [48–53].

### Study and patients’ characteristics

The main characteristics of the included studies are summarized in Table 1. From the 25 studies, 23 presented the registry number, with 16 international and 7 local registries. In eight of them, the registration was done after the beginning of recruitment, in one of them the recruitment period was not mentioned [16], and the protocols of five studies could not be retrieved for the registration date to be checked [16, 50, 54–56]. In sixteen studies, there were amends in the protocol after the registration [7, 18, 19, 48, 51–53, 57–65]

**Table 1:** Main characteristics of included studies

Reference	Trial registration number	Clinical trial blinding type	Setting/location and number of centers	Recruitment period	COVID-19 diagnosis method and % confirmed	Disease severity	Other inclusion criteria	Exclusion criteria
Ivermectin vs. placebo								
Ahmed et al. [16]	Bangladesh Medical Resource Council	Double-blinded	Hospital at Dhaka (Bangladesh) 1 center	Not clear	RT-PCR 100%	Not informed	Admitted to the hospital within the last 7 days; presence of fever (> 37,5ºC), cough and/or sore throat	Allergy to ivermectin or doxycycline, use of drug with potential for interaction with ivermectin or doxycycline, chronical illness, had received ivermectin or doxycycline in the last 7 days, pregnancy, breastfeeding, had participated in other RCT within the last month
Babalola et al. [54]	National Agency for Food and Drug Administration and Control in Lagos	Double blinded	Lagos University Teaching Hospital (Nigeria) 1 center	May to November, 2020	RT-PCR 100%	Asymptomatic or mild/moderate symptoms	NA	COVID-19 pneumonia or requiring MV, renal failure, thromboembolic complications or unconscious
Biber et al. [48]	NCT04429711	Double-blinded	Hotels in Tel-Aviv at Jerusalem and Ashkelon (Israel) 2 centers	May, 2020 to January, 2021	RT-PCR 100%	Mild to moderate, not requiring O_2_ and asymptomatic cases	Not pregnant, up to 7 days of symptoms onset	Weight < 40 kg, known allergy to the drugs, unable to take oral medication, participation in other RCT, RT-PCR results in Ct value > 35 in first two consecutive tests
Buonfrate et al. [57]	NCT04438850	Double-blinded	Outpatients laboratory-confirmed COVID-19 during the study period (Italy) 4 centers	July, 2020 to May, 2021	RT-PCR 100%	Mild, not requiring hospitalization or O_2_ supplementation	Asymptomatic or oligosymptomatic disease	Pregnancy, breastfeeding, CNS disease, participants under dialysis, severe medication conditions with prognosis < 6 months, warfarin treatment, antiviral/chloroquine phosphate/hydroxychloroquine treatment
Chaccour et al. [59]	NCT04390022	Double-blinded	University Clinical of Navarra (Spain) 1 center	July to September, 2020	RT-PCR 100%	Non-severe	< 72 h of cough or fever	Positive IgG, comorbidities considered risk factors for severe disease or COVID-19 pneumonia
Chachar et al. [80]	NCT04739410	Open label	Fatima Memorial Hospital at Lahore (Pakistan) Number not clear	May to June, 2020	RT-PCR 100%	Mild	NA	Known severe allergic reaction to ivermectin, pregnancy, breastfeeding, severe symptoms likely attributed to cytokine release storm, malignant disease, CKD, cirrhosis Child B or C
Beltran-Gonzalez et al. [51]	NCT04391127	Double-blinded	Hospital Centerio Miguel Hidalgo in the state of Aguascalientes (Mexico) 1 center	April to June, 2020	Confirmed or suspected % not informed	COVID-19 pneumonia (CO-RADS classification)	Suspected or confirmed COVID-19 cases as well as the pneumonia ATS criteria, hospitalization	Requirement of high O_2_ volumes, predictors of poor response to high-flow O_2_ nasal therapy or MV
Krolewiecki et al. [61]	NCT04381884	Open label, outcome assessor blinded,	Hospitals in the metropolitan area of Buenos Aires (Argentina) 4 centers	May to September, 2020	RT-PCR 100%	Mild to moderate	NA	Patients not requiring ICU admission, use of immunomodulators ≤ 30 days of recruitment, pregnancy, breastfeeding, poorly controlled comorbidities and known allergy to ivermectin
López-Medina et al. [19]	NCT04405843	Double-blinded	Colombian state’s health department electronic database (Colombia) 1 center	July to November, 2020	RT-PCR or antigen 100%	Mild to moderate	Symptoms began within the previous 7 days	Pregnancy, breastfeeding, hospitalized patients receiving high-flow O_2_ or MV, asymptomatic, severe pneumonia, received ivermectin within the previous 5 days, had hepatic dysfunction or liver function test results more than 1.5 × the ULN
Mohan et al. [62]	CTRI 2020/06/026001	Triple-blinded	COVID-19 facility at the National Cancer Institute, All India Institute of Medical Sciences, New Delhi (India) Number not clear	July to September, 2020	RT-PCR or antigen 79.6%	Non-severe: mild and moderate	NA	Pregnancy or lactation, known hypersensitivity to ivermectin, CKD with creatinine Cl < 30 mL/min, elevated transaminase levels, myocardial infarction or heart failure < = 90 days prior to enrolment, prolonged QTc, any other severe comorbidity or enrolment in concomitant RCT
Ravikirti et al. [65]	CTRI 2020/08/027225	Double-blinded	COVID-19 hospital (India) 1 center	August to October, 2020	RT-PCR or rapid antigen test 100%	Mild or moderate disease	NA	Severe disease, known allergy or adverse drug reaction to ivermectin, unwillingness or inability to provide consent to participate in the study, prior use of ivermectin during the course of this illness, pregnancy or lactation
Vallejos et al. [18]	NCT04529525	Double-blinded	Province of Corrientes (Argentina) Number not clear	August 19, 2020 to February 22, 2021	RT-PCR 100%	Not informed	≥ 18 years, residing in the province of Corrientes. If they are women of childbearing age, they should be using a contraceptive method of proven efficacy and safety. All individuals were to weigh at the time of inclusion ≥ 48 kg	Home O_2_ requirement; hospitalization for COVID-19 at the time of diagnosis; had a history of hospitalization for COVID-19; pregnancy; breastfeeding; known allergy to ivermectin or the components of ivermectin or placebo tablets; presence of mal-absorptive syndrome; presence of any other concomitant acute infectious disease; known history of severe liver disease; recent or expected need for dialysis; with participation in a research study that involved the administration of a drug < = 30 days
Reis et al. [7]	NCT04727424	Triple-blinded	12 public health clinics (Brazil)	June 2, 2020 to August 6, 2021	RT-PCR or rapid antigen test 100%	Mild-to-moderate COVID-19	≥ 18 years, outpatients, up to 7 days after symptom onset, and at least one high-risk criterion for progression of Covid-19 (age ≥ 50 years, diabetes, hypertension leading to the use of medication, cardiovascular disease, lung disease, smoking, obesity, organ transplantation, stage IV CKD or dialysis, immunosuppressive therapy, cancer)	Patients who needed hospitalization, severe terminal illness, RR > 28/min, SaO_2_ < 90% or < 93% on nasal oxygen therapy at 10 L/min, PaO_2_/FIO_2_ < 300 mmHg, use of the following medications in the last 14 days: monoamine oxide Inhibitors α-1 antagonists, sotalol, clonidine, Phosphodiesterase 5 inhibitors, Methyldopa, Prazosin, terasozin, doxazosin, antiretroviral agents, serotonin reception inhibitors; pregnancy or breastfeeding; surgical procedure or use of contrast planned up to 5 days after the last dose of the study medication; inability to give informed consent or adhere to the procedures proposed in the protocol; known hypersensitivity and / or intolerance to Ips, or taking medications contraindicated by Ips; inability to follow protocol-related procedures
Naggie S [52]	NCT04885530	Double-blinded	93 sites in USA	June 23, 2021 to February 4, 2022	RT-PCR or rapid antigen test 100%	Mild-to-moderate COVID-19	Sites verified eligibility criteria including age ≥ 30 years, confirmed SARS-CoV-2 infection within 10 days, and experiencing > 2 symptoms of acute COVID-19 for ≤ 7 days from enrollment. Symptoms included fatigue, dyspnea, fever, cough, nausea, vomiting, diarrhea, body aches, chills, headache, sore throat, nasal symptoms, and loss of sense of taste or smell	Hospitalization, study drug use within 14 days, or known allergy or contraindication to study drug. Ivermectin-specific exclusion criteria were end-stage kidney disease on renal replacement therapy, liver failure, decompensated cirrhosis, pregnancy, or breastfeeding
Ivermectin + SOC vs. SOC with no placebo								
Abd‐Elsalam et al. [58]	NCT04403555	Open‐label	Tanta and Assiut University Hospitals (Egypt) 2 centers	March to October, 2020	RT-PCR 100%	Mild to moderate	NA	Allergy or contraindications to the drugs used in the study, pregnant and breastfeeding mothers, and patients with cardiac problems
Bukhari et al. [49]	NCT04392713	Open‐label	Combined Military Hospital Lahore (Pakistan) 1 center	March to June, 2020	RT-PCR 100%	Either asymptomatic or mild/moderate symptoms	NA	Severe symptoms due to cytokine release syndrome, with uncontrolled comorbidities and immunocompromised states. Ivermectin allergy. Patients taking CYP3A4 inhibitors or inducers. Patients that had oxygen requirement equivalent to FiO_2_ ≥ 50%
Lim et al. [63]	NCT04920942	Open-label	20 government hospitals and a COVID-19 quarantine center (Malaysia)	May 31 to October 25, 2021	RT-PCR or antigen test 100%	Mild to moderate	≥ 50 years with at least 1 comorbidity, up to 7 days from symptom onset	Asymptomatic, supplemental oxygen requirement, SpO_2_ < 95% at rest, severe hepatic impairment, acute medical or surgical emergency, concomitant viral infection, pregnancy or breastfeeding, warfarin therapy, history of taking any antiviral drugs with reported activity against COVID-19 (favipiravir, hydroxychloroquine, lopinavir, and remdesivir) within 7 days before enrollment
Manomaipiboon et al. [50]	Navamindradhiraj University, Vajira Institutional Review Board no. 171/64	Double-blinded^c^	Faculty of Medicine, Vajira Hospital, Navamindradhiraj University, (Thailand) 1 center	September to November, 2021	RT-PCR 100%	Mild to moderate	18–80 years-old, within 72 h of a positive result or onset of symptoms	Pregnancy, breastfeeding, allergy or potential for a drug-drug interaction with ivermectin; previously treatment with ivermectin in the last 7 days; received herbal medicine; severe chronic illness; concurrent bacterial infection; severe symptoms; uncontrolled co-morbidities; immunocompromised status; unwilling to participate
Faisal et al. [55]	NA	NI	Shah Care Hospital (Pakistan) 1 center	April to May, 2020	RT-PCR 100%	Not informed	NA	Severe comorbidities, like diabetes mellitus, cardiovascular problems, CKD and O_2_ dependents
Okumuş et al. [60]	NCT04646109	Single-blinded	Research and Education Hospital (Turkey) 4 centers	May to September, 2020	RT-PCR 100%	Severe pneumonia	NA	< 18 years old, pregnancy, active breastfeeding, concurrent autoimmune disease, chronic liver or CKD, immunosuppression, SNP mutation in MDR-1/ABCB1 gene and/or haplotypes and mutations of the CYP3A4 gene
Podder et al. [56]	NA	Open-label	Debidwar Upazila (sub-district) Health Complex (Bangladesh) 1 center	May to July, 2020	RT-PCR 100%	Mild to moderate	NA	Known pre-existing hypersensitivity to ivermectin, pregnancy, breastfeeding and patients taking other antimicrobials or hydroxychloroquine, symptoms > 7 days or insufficient data
Shahbaznejad et al. [81]	Iran Registry of Clinical Trials 20111224008507N3	Double-blinded^c^	Hospitals of University of Medical Sciences (Iran) 2 centers	May to July, 2020	RT-PCR or symptoms + contact or chest CT 64%	Moderate to severe	NA	History of chronic liver and/or kidney disease, receipt of treatment with warfarin, angiotensin-converting enzyme inhibitor or angiotensin II receptor antagonist, acquired immunodeficiency, pregnancy or breastfeeding
La Rocha et al. [53]	NCT04407507	Double-blind	Guadalajara and Zapopan: Hospital Hispano (Mexico)	From 2020 July 21 to 2021 January	RT-PCR 100%	asymptomatic and mild COVID-19	> 18-year-old men and women diagnosed with SARS-CoV-2 infection by realtime polymerase chain reaction (RT–PCR) testing of nasopharyngeal swab samples. We considering viral load undetectable if the inferior limit was ≥ 40 copies/µL	Patients with moderate or severe COVID-19, 7 diagnosis of other respiratory infections, impaired liver function tests (> 5 times above the normal level of alanine aminotransferase or aspartate aminotransferase), history of recurrent urinary tract infections, pregnancy or nursing women, active participation in other clinical trials, and use of antibiotics, verapamil, cyclosporine A, trifluoperazine or antiparasitic treatment for a concomitant disease were excluded
Ivermectin vs. active comparator								
Galan et al. [82]	Brazilian Clinical Trial Database 8h7q82	Double-blinded^c^	General Hospital of Roraima (Brazil) 1 center	May to July, 2020	RT-PCR or IgM 100%	Severe^d^	Hospitalized by COVID-19	Patients < 18 years old, indigenous people, patients not fluent in Portuguese, unable to understand the objectives and methods of the study, critically ill patients who are not accompanied by legal representatives, those who reject participation in the study, patients with cardiac arrhythmia that include prolongation of the QT interval and previous use of the medication surveyed for more than 24 h
Niaee et al. [64]	Iran Registry of Clinical Trials 20200408046987N1	Double-blinded^c^	Public hospitals in Qazvin and Khuzestan(Iran) 5 centers	June to July, 2020	RT-PCR or symptoms + contact or chest CT 71%	Mild to moderate	NA	Children, severe immunosuppression, pregnant women, known allergic reaction to the intervention drugs, chronic kidney disease, malignancy, severe COVID-19 patients and indications that the patients were unable and/or unlikely to comprehend and/or follow the protocol

Reference	Randomized sample	Final sample	Age^a^	Ivermectin	Comparator	Funding
Ivermectin vs. placebo						
Ahmed et al. [16]	72	68	42	A1: Ivermectin 12 mg for 5 days; A2: Ivermectin 12 mg for 4 days	Placebo	Beximco Pharmaceutical Limited, Bangladesh
Babalola et al. [54]	63	62	44.1 ± 14.7	A1: Ivermectin 6 mg (given every 84 h) twice a week; A2: Ivermectin 12 mg (given every 84 h) for 2 weeks + SOC^e^	SOC^e^ + placebo	NI
Biber et al. [48]	116	89	35 (28–47)	Ivermectin 0.2 mg/kg for 3 days	Placebo	NI
Buonfrate et al. [57]	93	93	47 (31.0–58.0)	Single dose 54 ivermectin 600 μg/kg plus placebo for 5 days (arm B); single dose ivermectin 1200 μg/kg for 5 days (arm C)	Placebo	Italian Ministry of Health
Chaccour et al. [59]	24	24	NI	Ivermectin 0.4 mg/kg (single oral)	Placebo	ISGlobal, Barcelona Institute for Global Health and Clínica Universidad de Navarra
Chachar et al. (2020) [80]	50	50	41.8 ± 15.7	Ivermectin 12 mg (3 tablet in stat, 12 h and 24 h)	Placebo	NI
Beltran-Gonzalez et al. [51]	108	106	53.8 ± 16.9	Ivermectin 0.2 mg/kg for 4 days	A1: Placebo A2: HCQ^c^	NI
Krolewiecki et al. [61]	45	45	NI	Ivermectin 0.6 mg/kg/day for 5 consecutive days with either breakfast or lunch at approximately 24 h intervals	Placebo	IP-COVID-19–625, Agencia Nacional de Promoción de la Investigación, el Desarrollo Tecnológico y la Innovación, Argentina and Laboratorio ELEA/Phoenix, Argentina
López-Medina et al. [19]	476	398	37 (29—47.7)	Ivermectin 0.3 mg/kg for 5 days	Placebo	Centro de Estudios en Infectología Pediátrica (grant ScDi823)
Mohan et al. [62]	157	152^b^	35.3 ± 10.4	A1: Ivermectin 12 mg (single dose);A2: Ivermectin 24 mg (single dose)	Placebo	Science and Engineering Research Board, Department of Science and Technology, Government of India
Ravikirti et al. [65]	115	112	52.5 ± 14.7	Ivermectin 12 mg (single dose) for 2 days	Placebo	Ivermectin tablets were procured from the learning resource allowance of the principal investigator Placebo tablets were provided by Sun Pharma Pvt. Ltd
Vallejos et al. [18]	501	501	42.49 ± 15.51	Ivermectin^f^ + SOC^g^	Placebo + SOC^g^	NI
Reis et al. [7]	679	679	49 (38–57)	Ivermectin^h^ + SOC^i^	Placebo + SOC^i^	Bill and Melinda Gates Foundation (INV-019641)
Naggie [52]	1591	1591	48 years ± 12	Ivermectin 400 µg/kg for 3 days	Placebo	National Center for Advancing Translational Sciences (NCATS) (3U24TR001608-05W1)
Ivermectin + SOC vs. SOC with no placebo						
Abd‐Elsalam et al. [58]	164	164	NI	Ivermectin 12 mg (single dose) for 3 days + SOC^j^	SOC^k^	NI
Bukhari et al. [49]	100	86	NI	Ivermectin 12 mg (single dose) + SOC^k^	SOC^l^	NI
Faisal et al. [55]	100	100	NI	Ivermectin 12 mg once a day for 5 days + azithromycin + SOC^l^	Azithromycin + SOC^m^	NI
Okumuş et al. [60]	66	60^a^	NI	Ivermectin 0.2 mg/kg for 5 days + SOC^m^	SOC^n^	Afyonkarahisar Health Science University Scientific Research project
Podder et al. [56]	82	62	39.2 ± 12.1	Ivermectin 0.2 mg/kg (single dose) + SOC^n^	SOC^g^	Self-financiated
Shahbaznejad et al. [81]	70	69	46.4 ± 22.5	Ivermectin 0.2 mg/kg + SOC^o^	SOC^f^	NI
Manomaipiboon et al. [50]	72	72	48.57 ± 14.8	Ivermectin 12 mg per day, por 5 days	SOC^o^	Navamindradhiraj University (grant 171/64)
Lim et al. [63]	500	490	62.5 ± 8.7	0.4 mg/kg body weight daily for 5 days + SOC	SOC^p^	NI
La Rocha et al. [53]	66	56	Placebo36.4 (13) IVM 40 (15.4)	12 mg per day ivermectin tablets or placebo for 3 days + SOC^q^	Placebo + SOC^q^	Biomédica para el Desarrollo de Fármacos S.A. de C.V
Ivermectin vs. active comparator						
Galan et al. [82]	167	167	53.4 ± 15.6	A1: Ivermectin (14 mg once at day 0 + 1 placebo tablet at day 0, and once daily from day 1 to day 2, + 1 placebo tablet daily from day 3 to 4, total dose 42 mg; A2: Ivermectin (14 mg once at day 0 + 1 placebo tablet at day 0, and once daily from day 1 to day 2, + 1 placebo tablet daily from day 3 to 4, total dose 42 mg)	A1: Hydroxychloroquine A2:CQ diphosphate + HCQ sulfate	NI
Niaee et al. [64]	180	180	56 (45–67)	A1: Ivermectin 0,2 mg/kg (1 tablet, single dose); A2: Ivermectin 0,2 mg/kg (3 tablet in day 1, 3 and 5); A3: Ivermectin 0,4 mg/Kg (2 tablets per day, single dose); A4: Ivermectin (0.4 mg/kg, 4 tablets in day 1; 0.2 mg/kg, 4 tablets in day 3; 0.2 mg/kg, 4 tablets in day 5)	HCQ	Research deputy of Qazvin University of Medical Sciences and Science and Technology Park, Qazvin, Iran

Numbers are presented as average ± standard deviation or median (interquartile range)

*A* arm, *ALT* alanine aminotransferase, *AST* aspartate aminotransferase, *ATS* American Thoracic Society, *CKD* chronic kidney disease, *CKD-EPI* Chronic Kidney Disease Epidemiology Collaboration, *Cl* clearance, *CO-RADS* COVID-19 Reporting and Data System [74], *CNS* central nervous system, *Ct* cycle threshold, *CTRI* Clinical Trials Registry-India, *GFR* glomerular filtration rate, *HCQ* hydroxychloroquine, *ICU* intensive care unit, *IP* Pegylated interferon, *MV* mechanical ventilation, *N* no, *NA* not applicable, *NCT* National Clinical Trial Number, *NI* not informed, *O*_*2*_ oxygen, *QTc* corrected Qt interval, *RCT* randomized controlled trial, *SNP* single nucleotide polymorphism, *SOC* standard of care, *Y* yes, *ULN* upper limit of normality

^a^The final sample for adverse effects is different to the final sample for our primary outcomes

^b^This arm was not included in the meta-analysis

^c^Although there is no placebo, the researchers described that patients were blinded

^d^Defined as: dyspnea, tachypnea, peripheral oxygen saturation < 93%, PaO_2_/FiO_2_ ratio < 300 or pulmonary infiltrate > 50%

^e^Not informed

^f^Those weighing up to 80 kg received 2 tablets of 6 mg (mg) each at inclusion and another 2 tablets of 6 mg each 24 h after the first dose (total 24 mg). Those weighing more than 80 kg and up to 110 kg received 3 tablets of 6 mg each at inclusion and another 3 tablets of 6 mg each 24 h after the first dose (total 36 mg). Those weighing more than 110 kg received 4 tablets of 6 mg each at inclusion and another 4 tablets of 6 mg each 24 h after the first dose (total 48 mg)

^g^Antipyretics, cough suppressants, and capsule doxycycline 100 mg every 12 h for seven days

^h^400 μg per kilogram of body weight once daily for 3 days

^i^SOC: standard care for Covid-19 provided by health care professionals in Brazil

^j^Lopinavir/ritonavir

^k^Paracetamol, oxygen, fluids (according to the condition of the patient), empiric antibiotic, oseltamivir if needed (75 mg/12 h for 5 days), and invasive

mechanical ventilation with hydrocortisone for severe cases if PaO_2_ less than 60 mm Hg, O_2_ saturation less than 90% despite oxygen or noninvasive ventilation, progressive hypercapnia, respiratory acidosis (pH < 7.3), and progressive or refractory septic shock

^l^Oral vitamin C 500 mg once daily, oral vitamin D3 200,000 IU once weekly, and oral paracetamol 500 mg SOS

^m^Paracetamol (500 mg if needed), Vit C (500 mg once a day for 15 days), zinc (20 mg twice a day for 15 days) and Vit D (injection PO 200,000units once) supplements

^n^Hydroxychloroquine (2 × 400 mg loading dose followed by 2 × 200 mg, po, 5 days), favipiravir (2 × 1600 mg loading dose followed by 2 × 600 mg maintenance dose, po, total 5 days) and azithromycin (500 mg first day loading dose, followed by 250 mg/day, po, total 5 days)

^o^Favipiravir or andrographolide; corticosteroids; cetrizine; paracetamol

^p^Symptomatic treatment

^q^Acetaminophen 500 mg four times a day for 14 days

All studies had parallel designs. Twelve studies took place in Asia (Pakistan n = 03; India n = 02; Bangladesh n = 02; Iran n = 02; Turkey n = 01; Israel n = 01;Malasia = n = 01); eight were in South America (Argentina n = 02; Colombia n = 01; Mexico n = 02; Brazil n = 02); one in North America (USA = 01); two in Africa (Egypt n = 01; Nigeria n = 01) and two in Europe (Spain n = 01; Italy n = 01). The number of centers included in each study ranged from one to 93, from hospitals to outpatient clinics (Table 1).

Included studies provided a total of 6310 subjects. Of those, 50.5% were men. From the 20 studies which reported disease severity at baseline (n = 4030), 56 patients were reported to have between asymptomatic to mild COVID-19 (1.4%) 1028 (25.5%) patients had mild; 560 (13.9%) moderate; 387 (9.6%) severe COVID-19; and 1999 (40.6%%) patients were reported to have between mild to moderate disease severity.

In 14 studies ivermectin was compared to placebo, in nine ivermectin was associated with SOC and compared to SOC alone, and in two ivermectin was compared to an active drug. SOC definition varied considerably among them, possibly including antibiotics, antivirals, hydroxychloroquine, vitamins and mineral supplements (Table 1). Ivermectin doses ranged from 0.4 mg/kg (single oral) to 12 mg (3 tablets 0, 12 and 24 h), for five to 90 days. Follow-up ranged from 7 to 90 days.

From the 25 studies, only 16 reported the funding source, five of them funded by the pharmaceutical industry [16, 53, 59, 65].

### Quality assessment

The risk of bias for each study is depicted in Additional file 1: Fig. S1 and Additional file 2: Fig. S2. From the fourteen studies which assessed mortality comparing ivermectin to placebo, four had high risk of bias, six had some concerns and five had low risk of bias. From the seven studies in which ivermectin + SOC was compared to SOC, five studies had high risk of bias and three had some concerns. Overall, for this outcome, when taking into account only studies which reported any events, two had high risk of bias, four had some concerns and three low risk of bias.

From the fourteen studies which assessed the mechanical ventilation support end point, in ten ivermectin was compared to placebo. Two of those had high risk of bias, four some concerns and four low risk of bias. When ivermectin associated to SOC was compared to SOC, one study had high risk of bias and three some concerns. Of the seven with events, two had low risk of bias, four had some concerns and one had high risk of bias.

The main limitations of those with high risk were lack of concealment of the randomization sequence and allocation when distributing patients to the study groups, lack of adequate blinding of patients, treating physicians and outcome assessors, in addition to the relevant number of subjects’ exclusion after randomization.

### Primary outcomes

Table 2 shows the summary of findings (SOF) table with the GRADE classification of the quality of evidence for the primary outcomes.

**Table 2:** Summary of findings (SOF) table for the primary outcomes

Outcome	Study population	Relative effect RR (95% CI)	Certainty of the evidence (GRADE)
Mortality	6048 patients^a^ (24 studies)	(RR = 0.76; 95%CI: 0.52–1.11)	⊕ ⊕ Low
Mechanical ventilation requirement	5270 patients^b^ (153 studies)	(RR = 0.74; 95%CI: 0.48–1.16)	⊕ ⊕ Low

The body of evidence was graded as “low” due to serious risk of bias and imprecision

*GRADE* Grading of Recommendations Assessment, Development and Evaluation, *RR* relative risk

^a^6288 patients randomized, but 6048 included in the analysis

^b^5405 patients randomized, but 5270 included in the analysis

**Fig. 2:**
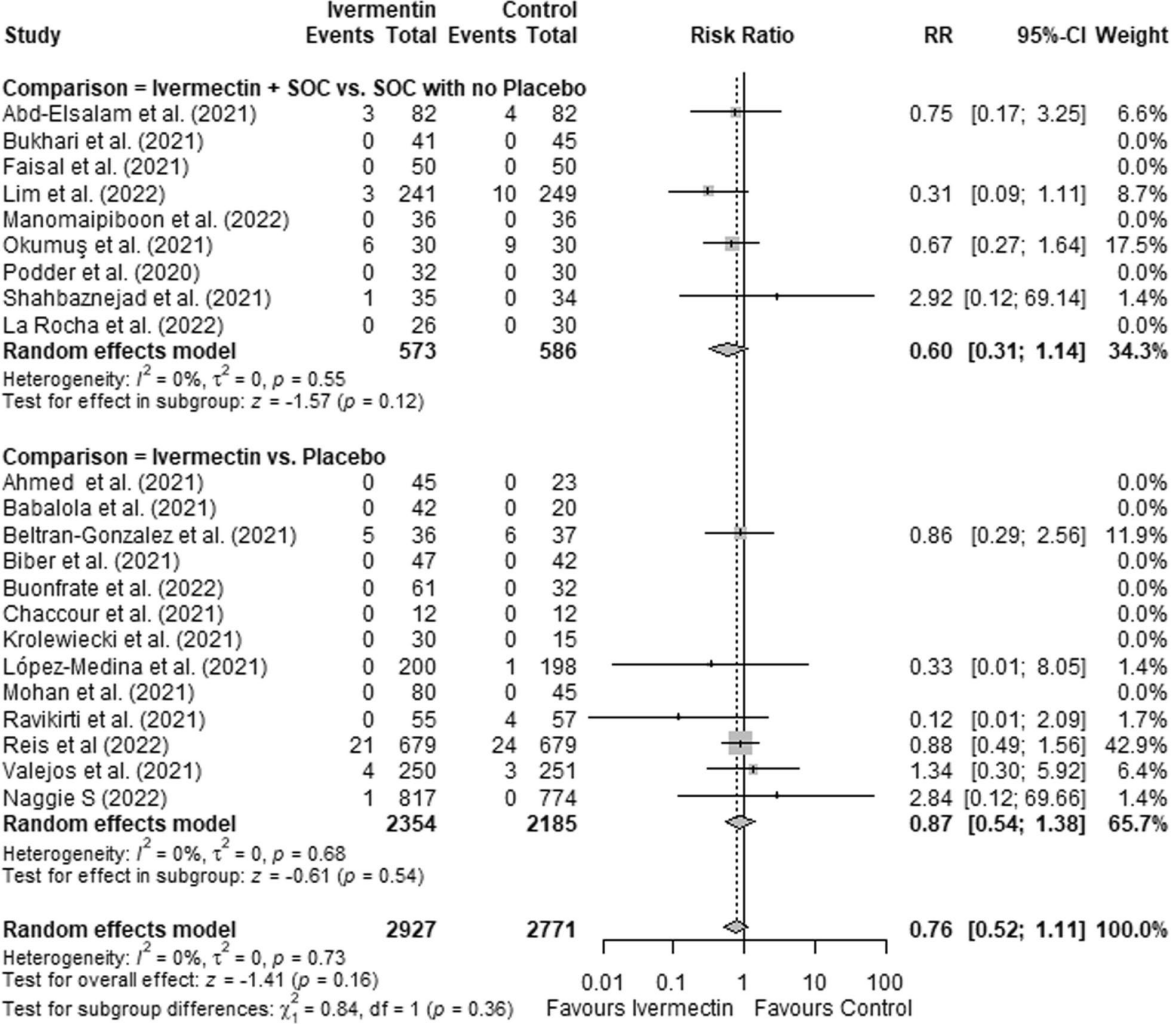
Forest plots showing the risk of mortality in patients who took ivermectin compared to controls, stratified by placebo or other drugs. *RR* relative risk. Asterisk indicates that this study had two control groups, one with placebo and the other with another drug. We included in the pooled analysis only the comparator arm which used placebo

**Fig. 3:**
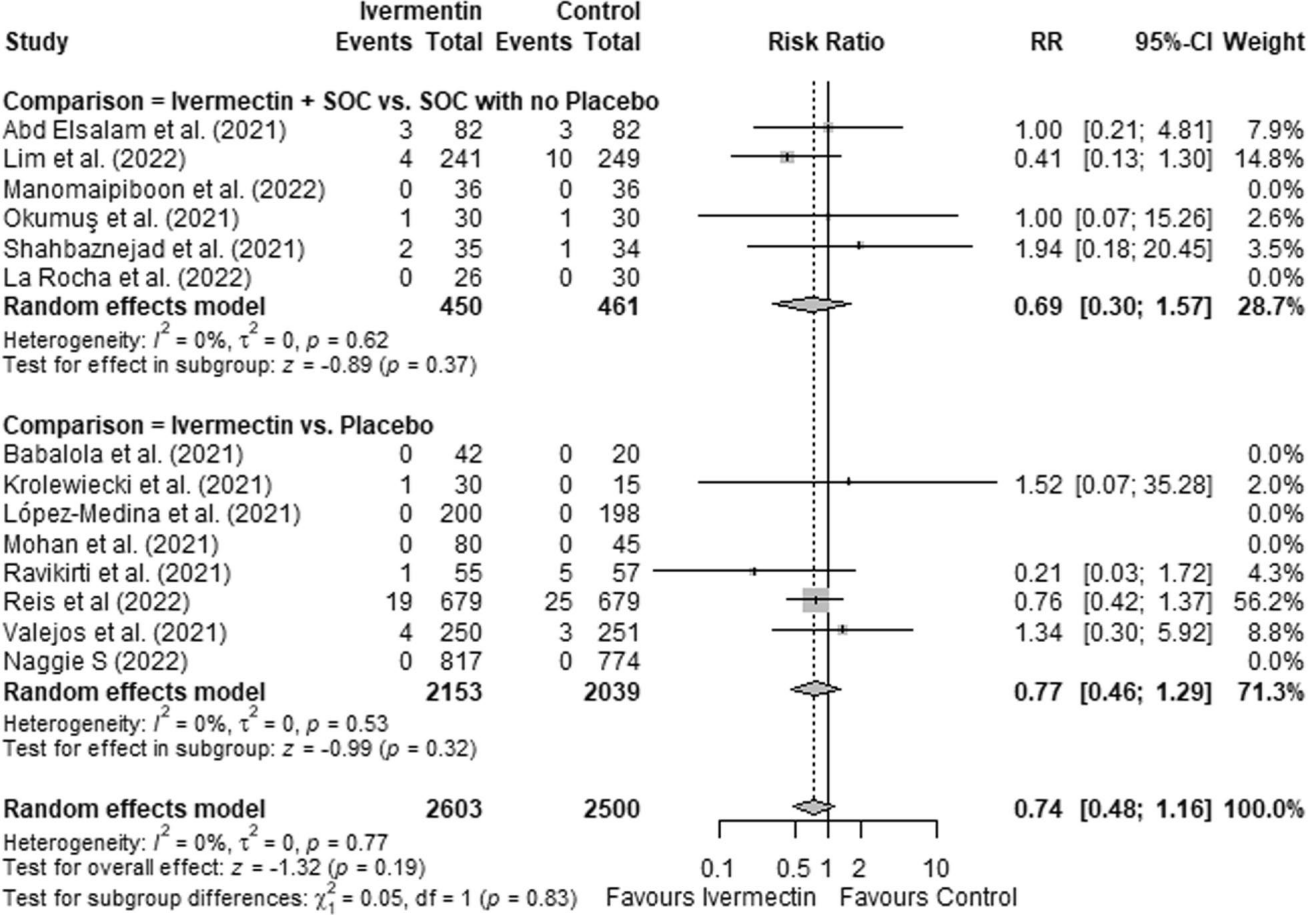
Forest plots showing the risk of mechanical ventilation requirement in patients who took ivermectin compared to controls, stratified by placebo or other drugs. *RR* relative risk

Treatment with ivermectin did not show significant effect on mortality (RR = 0.76; 95%CI: 0.52–1.11; *I*^2^ = 0%) (Fig. 2) or the need for invasive ventilation (RR = 0.74; 95% CI 0.48–1.16; *I*^2^ = 0%) (Fig. 3), with no difference whether ivermectin was compared to placebo or whether ivermectin associated with SOC was compared to SOC (p = 0.39 for mortality and p = 0.83 for ventilation).

Visual inspection of the funnel plots (Additional file 3: Fig. S3A, Additional file 4: Fig. S3B), Eggers test (p = 0.77 for mortality and p = 0.71 for mechanical ventilation) and “Trim and fill” method for mortality (RR = 0.76; 95%CI: 0.52–1.11) and for ventilation (RR = 0.71; 95%CI: 0.46–1.09) showed no evidence of publication bias, even those results comes from small sample sizes.

**Fig. 4:**
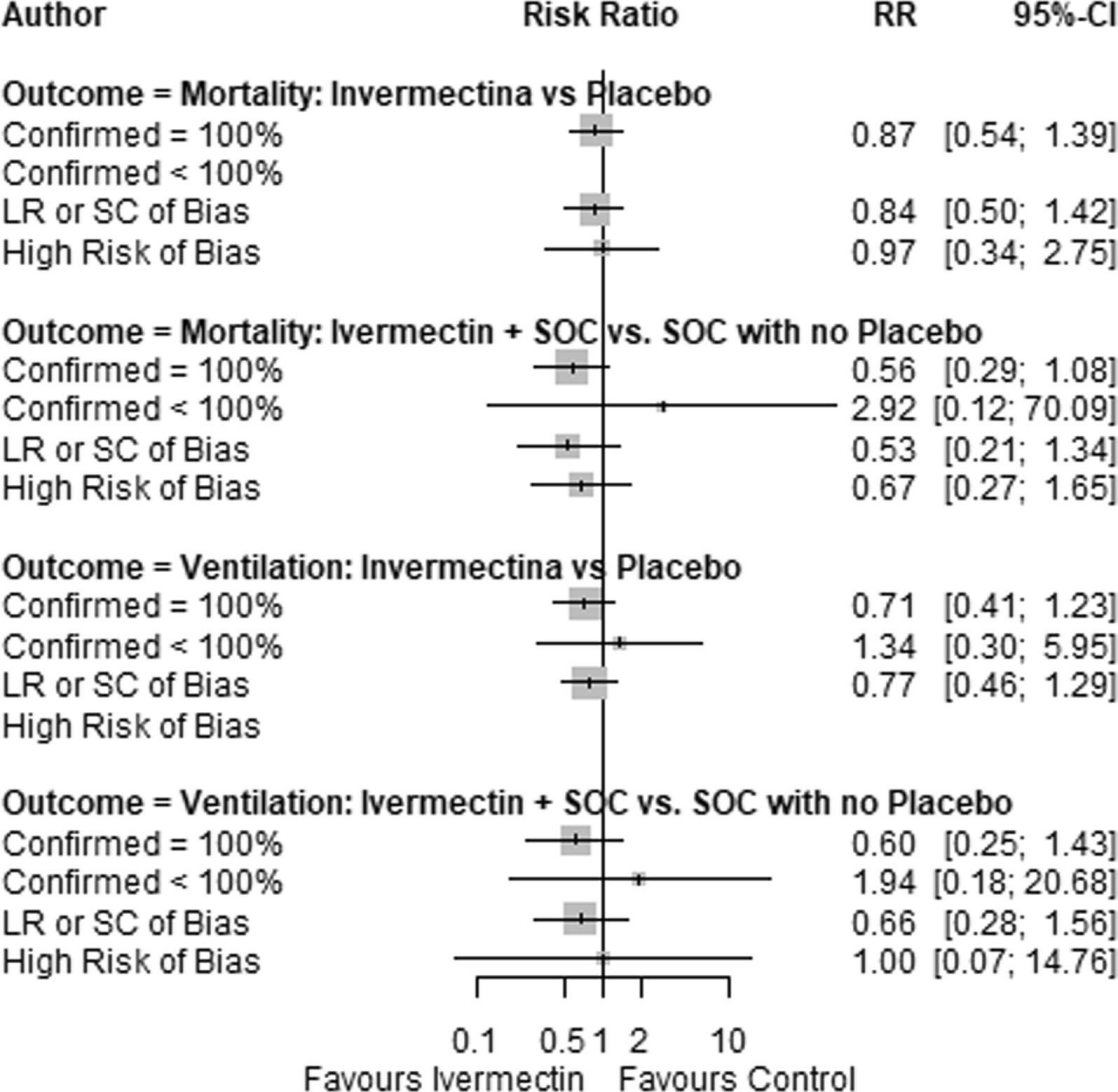
Forest plots showing sensitivity analysis of mortality and mechanical ventilation according to the percentage of confirmed COVID-19 patients and risk of bias. *RR* relative risk

Results did not differ substantially in sensitivity analysis taking into account the risk of bias and the percentage of confirmed individuals of the included studies (Fig. 4) and by removing each study one-by-one or excluding simultaneously studies with extreme results (Additional file 5: Fig. S4).

### Secondary outcome

Overall, ivermectin did not show evidence of association with the occurrence of adverse effects when compared with control (RR = 1.07; 95%CI: 0.84–1.35; *I*^2^ = 53%) (Additional file 6: Fig. S5), with no difference between subgroups (p = 0.27).

## Discussion

We conducted a comprehensive search on the impact of ivermectin for the management of patients with COVID-19 and observed that ivermectin does not have an effect in reducing mortality or mechanical ventilation in patients with COVID-19. Despite the low quality of evidence, this effect was consistent when comparing ivermectin vs. placebo, and ivermectin associated with SOC vs. SOC, as well as in sensitivity analysis. Additionally, there was very low quality of evidence of no increase in risk of adverse effects.

Despite not being recommended in current COVID-19 guidelines by WHO and IDSA [4, 30] the prevalence of self-medication during COVID-19 course was high, and ivermectin was one of the medications commonly used, as shown in a recent systematic review [66]. This may be related to self-medication, misinformation in the media, science denialism and low access to health services combined with the low cost of ivermectin, and the belief that it has a safe adverse effect profile [66, 67]. In Brazil, for example, the Ministry of Health included the medication in its COVID-19 guidelines. Up to August 2021, estimates from the Brazilian Parliamentary Commission of Inquiry showed that only one pharmaceutical company sold more than 83 million US dollars in ivermectin [68, 69].

Living systematic reviews may have changed this scenario, as they are supposed to incorporate all new relevant evidence as they become available [70]. Nonetheless, with the overwhelming number of studies published in COVID-19 pandemic area, keeping the living reviews updated is a challenge difficult to overcome. This challenge is even more complex when the living reviews propose to assess different comparisons with multiple drugs and evolving SOC. Ivermectin has been the subject of two systematic reviews. The British Medical Journal's living review was last updated not so recently, in April 2021, and suggested a possible reduction in mortality in patients who used ivermectin, when compared to standard of care (RR 0.31 95% CI 0.14–0.072). The authors highlighted the fact that data was limited by extremely few events, leading to very serious imprecision, and serious risk of bias [28]. The other living review, by the Pan American Organization, has been recently updated in December 2021, and analyzed the evidence from 14 studies. The authors reported that pooled estimates suggested mortality reduction with ivermectin (RR 0.50 95% IC 0.29–0.87), an effect that was no longer apparent when a subgroup analysis of the three studies classified as low risk of bias was performed (RR 0.96 95%CI 0.58–1.59) [71]. These two living reviews were not updated after Elgazzar et al., Samaha et al. and Pott-Junior et al. studies were retracted. We reckon that another update must also take into account the fact that studies that compared ivermectin with hydroxychloroquine may now be clinically inappropriate, and therefore should not be kept in the pooled analysis studies, as evidence of harm with the use of hydroxychloroquine is now robust [38].

Differently from our results, a recent review has found substantial differences in the results of studies with or without important methodological limitations, highlighting that important benefits associated with ivermectin were based on potentially biased results [72]. Because this review was mainly interested in investigating “bias as a source of inconsistency”, as stated in the review title, the authors included in the pooled analysis Elgazzar et al. retracted trial (and studies which were retracted after the publication of the systematic review), as well as studies in which ivermectin was compared to other drugs, such as hydroxychloroquine [64, 73]. We strongly believe the best body of evidence now available should not include such studies. Additionally, several studies were published after the publication of this review [7, 45, 52, 53].

In the present study, the certainty of the evidence on mortality and need for mechanical ventilation was ranked as low (GRADE) due serious concerns about risk of bias and imprecision. Methodological limitations were mainly due to lack of adequate blinding of patients and outcome assessors and high number of losses after randomization. Additional concerns included the fact that some studies were not pre-registered prior to enrolling patients, others had the protocol modified, and the majority did not report the funding source, although three of them were sponsored by pharmaceutical companies. However, as mortality and mechanical ventilation are hard endpoints, and our findings were negative, those sources of bias might not have had a great impact on these outcomes. Instead, it could have been influential for assessing adverse effects. In fact, studies usually assess overall adverse effects, without separating them according to severity, and this is a limitation addressing this outcome. For example, in Okumus et al., while patients who took ivermectin had serious neurological adverse effects which require drug discontinuation, the control group had only nausea, vomiting or two-fold increase in alanine transaminase. None of these side effects were severe enough to require termination of treatment in the control group [58].

Another limitation is the low event rate. Among the 22 studies that could be assessed for mortality, 11 did not have any events. COVID-19 severity varied among the different studies, but the majority of them included patients with mild to moderate disease. Therefore, mortality is expected to be very low in this context. The same applies for mechanical ventilation requirement.

One strength of the present review is to have applied strict methodological criteria, to have performed a broad search in several databases, and to be comprehensive, analyzing not only studies comparing the drug to placebo, but also those in which ivermectin associated with SOC was compared to SOC, in a stratified analysis. This is different from a recent Cochrane review, with the last search performed in May 2021, which selected only placebo-controlled studies. Only two studies were included in the pooled analysis to assess mortality and mechanical ventilation requirement [29]. As aforementioned, a recent network meta-analysis has shown how different statistical approaches (random vs. fixed) lead to different results on the effects of ivermectin on viral clearance [15]. This shows how misleading results may be when inappropriate methods are used. Random effects model is more appropriate in this context, as studies included patients with heterogeneous disease severity and the management was different among the different studies. This could be observed by the several definitions of standard of care. As we should not assume a common effect size to all studies included, and the goal of the analysis is to generalize to a range of scenarios, a random effect approach is recommended [74].

The urgent demand for treatment options for COVID-19 has created the need for randomized clinical trials. Scientists tested several approved drugs against the disease, “throwing every already-approved drug” [9], and the rush to conduct those trials led to conduct and publication of studies with varied quality and important methodological limitations. This “provided fertile ground for even poorly evidenced claims of efficacy to be amplified, both in the scientific literature and on social media” [20]. This is very problematic, as results from studies with high risk of bias were quickly widespread in clinical practice and public policy and SOC were also adopted in a rush in different countries. Even worse, different governments were reluctant to change their protocols after the evidence had shown that some drugs should not be used. In Brazil, for example, a huge polarization and politicization disseminated the SOC supported by the Brazilian President, known as “kit COVID-19” which included hydroxychloroquine, ivermectin and azithromycin [8]. Consequently, studies have included varied and clinically inappropriate options as they defined their comparators as SOC.

For example, months after evidence that hydroxychloroquine may increase the risk of death was available [75] a research protocol of Beltran-Gonzalez et al. study was registered, in Brazil [50]. With regard to antibiotics as SOC in patients with COVID without evidence of bacterial pneumonia (for example doxycycline or azithromycin), the World Health Organization advised against the practice in May 2020; still, two studies which started recruitment at that month kept antibiotics in their definition of SOC [56–60].

There are ethical considerations in this regard, exposing patients to harm and, in the case of antibiotics, contributing to the emergence of antibiotic resistance [70], which is a major issue worldwide. In a letter to the editor about Podder et al., Meneses pointed out important ethical issues. He observed that the authors mentioned approval of their study by the director of the health center, but apparently there was methodological and ethical evaluation by an institutional board [76].

Niaee et al., which included hydroxychloroquine in the comparison arms and was the main study which has shown benefits of ivermectin in clinical outcomes after the aforementioned retractions [62], has been recently questioned. An editorial note was published in October 2021, reporting concerns about various aspects of the study, including possible problems in the randomization of participants [20]. This raises concerns that flawed evidence in studies with ivermectin or other drugs may impact in systematic reviews.

Our meta-analysis was innovative for using the Living Overview of Evidence database (L.OVE, issued by Epistemonikus) for a comprehensive search, in addition to the traditional search. L.OVE is a digital tool that compiles articles from several databases, including preprint databases, kept up to date through computational algorithms [77]. A previous analysis has shown that it may be more efficient than the traditional search [78]. Consequently, in terms of databases, our search was broader than other systematic reviews on the topic, and the tool made it easier to update the search regularly. Furthermore, we tried to minimize potential biases in the review process by following the methods recommended by the Cochrane Collaboration [31] and set out in our published PROSPERO protocol [32], which provides transparency in the review process. Additionally, we presented a summary of findings table with GRADE results and assessment, in accordance with the new standards required by Cochrane.

In a recent publication, the authors reflect that besides the retracted studies, several other studies which claim a benefit for ivermectin may be similarly fraught. They highlighted unexplainable mismatches between trial registry updates and published patient demographics and timelines that are not consistent with the veracity of the data collection [20]. Therefore, it is of utmost importance for authors of systematic reviews, before following strict methodological criteria, to keep updated with possible new study retractions. Our sensitivity analysis did not show any difference in the point estimates when individual studies were removed, so we do not expect large changes in point estimates.

Thousands of supporters, many of them anti-vaccine activists, have continued to vigorously campaign for ivermectin use, claiming that the real evidence is being ignored. In the context of misinformation infodemics, some sites have published systematic reviews with meta-analysis on the effectiveness of the use of ivermectin in COVID-19 outcomes (https://ivmmeta.com and Home—FLCCC|Front Line COVID-19 Critical Care Alliance (covid19criticalcare.com) Most reviews have not undergone peer review, do not show the criteria used in the selection of RCT's, do not present records and statistical criteria for evaluating the effect and heterogeneity between studies. According to Roman et al., these sites contribute to misinformation of patients, their families, the general population and health professionals who cannot critically analyze scientific studies.

We believe this transparent and thorough summary may contribute to disseminate truthful evidence. Despite the limitation in the analysis of adverse effects, previous studies list some serious adverse effects, such as toxidermias, encephalopathies, confusional disorders [79]. Associated with the lack of clinical benefit, this should be considered when managing patients with COVID-19.

## Conclusion

The evidence suggests that ivermectin does not reduce mortality risk and the risk of mechanical ventilation requirement. Although we did not observe an increase in the risk of adverse effects, the evidence is very uncertain regarding this endpoint.

## Supplementary Information

**Additional file 1. Figure S1.** Risk of bias for randomized controlled trials which assessed mortality, stratified by comparator.

**Additional file 2. Figure S2.** Risk of bias for randomized controlled trials which assessed invasive mechanical ventilation support, stratified by comparator.

**Additional file 3. Figure S3A.** Funnel plot for the risk of mortality.

**Additional file 4. Figure S3B.** Invasive mechanical ventilation support.

**Additional file 5.** Forest plots of sensitivity analysis of mortality and invasive mechanical ventilation support.

**Additional file 6.** Forest plots showing the risk of adverse effects in patients who took ivermectin compared to controls, stratified by placebo or other drugs. RR: relative risk.

**Additional file 7.** Search strategy.

## Data Availability

The datasets analyzed during the current study are available from the corresponding author on reasonable request.

## Abbreviations

COVID-19: Coronavirus disease 2019
Decs: Descritores em Ciências da Saúde
IDSA: Infectious Disease Society of America
LILACS: Latin American and Caribbean Health Science Information
L.OVE: Living Overview of the Evidence
MeSH: Medical Subject Headings
PRISMA: Preferred Reporting Items for Systematic Reviews and Meta-Analyses
RRs: Risk ratios
RCT: Randomized controlled trials
SARS-CoV-2: Severe acute respiratory syndrome coronavirus 2
SOC: Standard of care
WHO: World Health Organization
