# Supplementary figures and images for: Systematic review and meta-analysis of ivermectin for treatment of COVID-19: evidence beyond the hype
Source: BMC Infect Dis. 2022 Jul 23;22:639. doi: 10.1186/s12879-022-07589-8 (PMC9308124; doi:10.1186/s12879-022-07589-8)

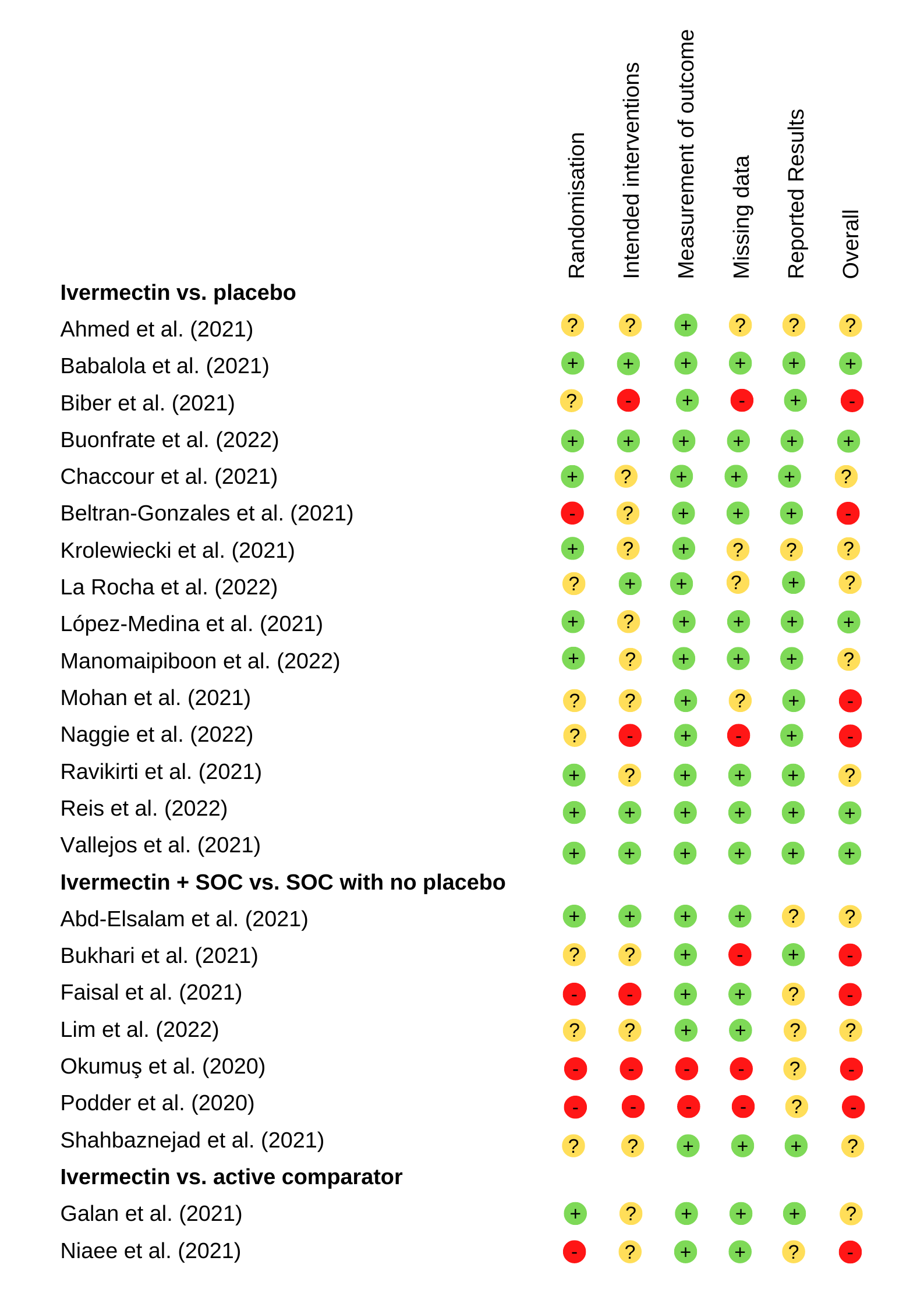

Supplement: Supplementary file 1 — Additional file 1. Figure S1. Risk of bias for randomized controlled trials which assessed mortality, stratified by comparator. [file 12879_2022_7589_MOESM1_ESM.png]

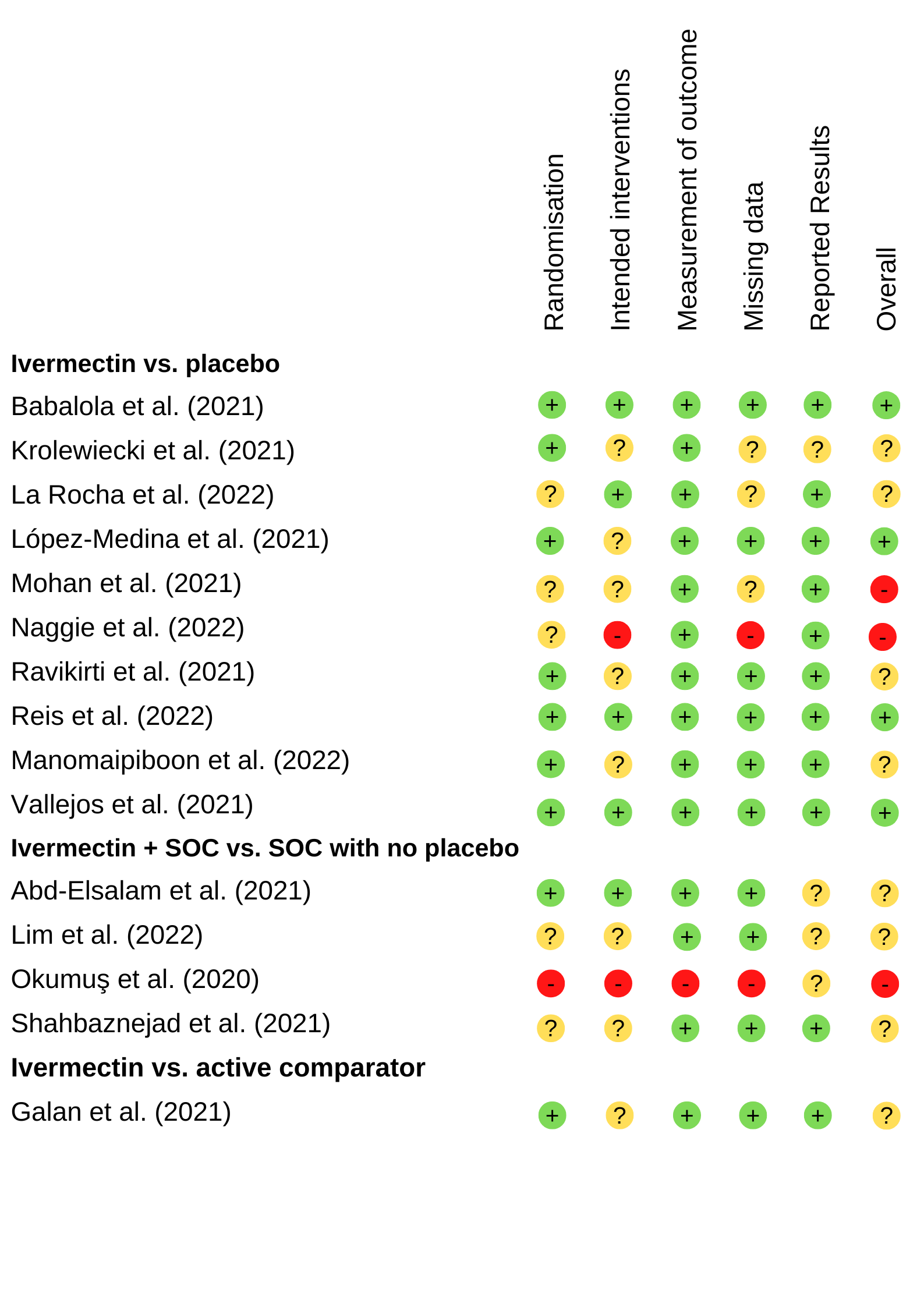

Supplement: Supplementary file 2 — Additional file 2. Figure S2. Risk of bias for randomized controlled trials which assessed invasive mechanical ventilation support, stratified by comparator. [file 12879_2022_7589_MOESM2_ESM.png]

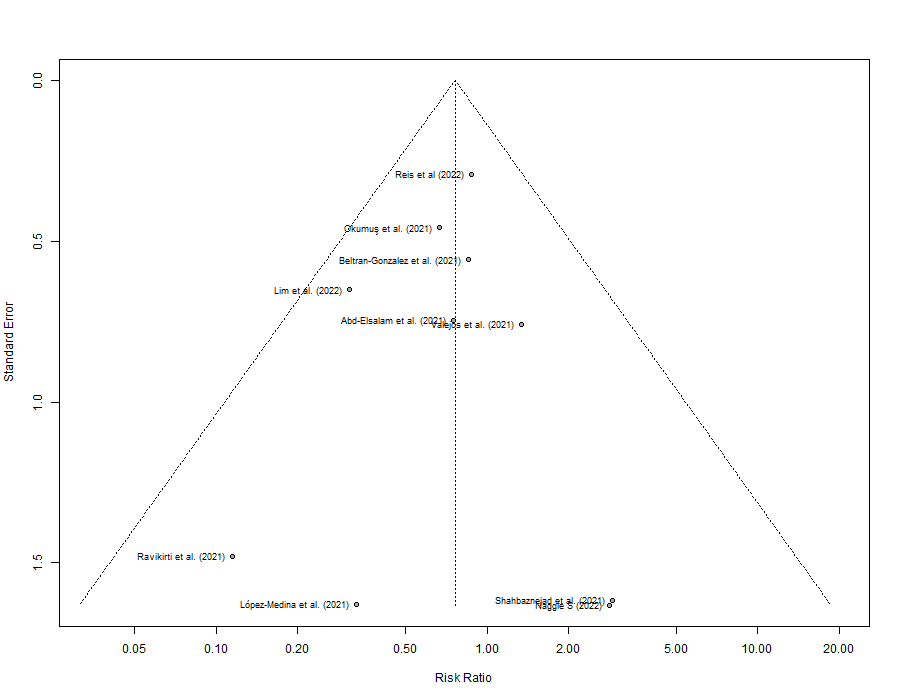

Supplement: Supplementary file 3 — Additional file 3. Figure S3A. Funnel plot for the risk of mortality. [file 12879_2022_7589_MOESM3_ESM.png]

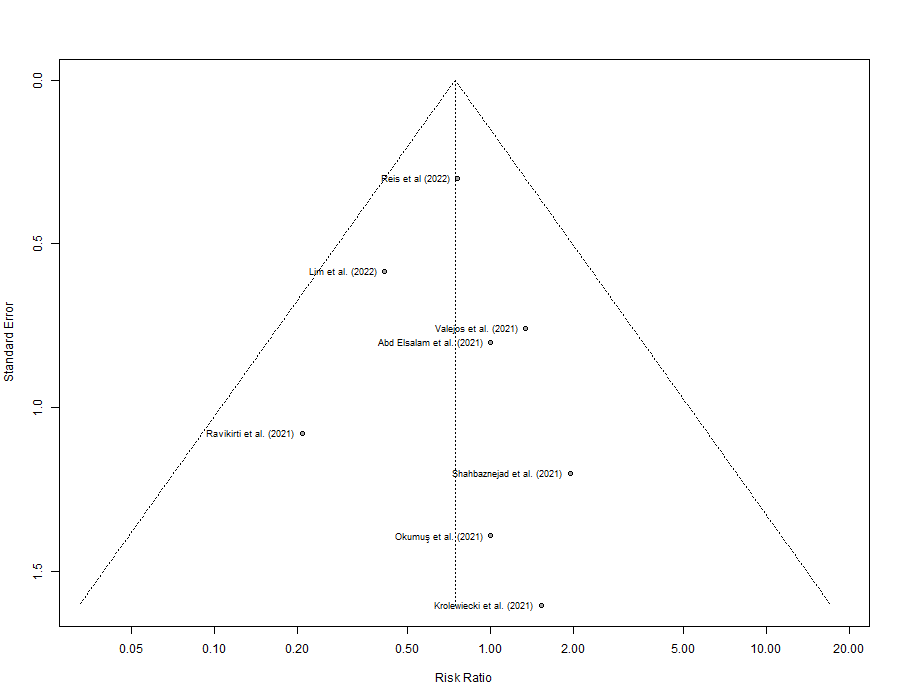

Supplement: Supplementary file 4 — Additional file 4. Figure S3B. Invasive mechanical ventilation support. [file 12879_2022_7589_MOESM4_ESM.png]

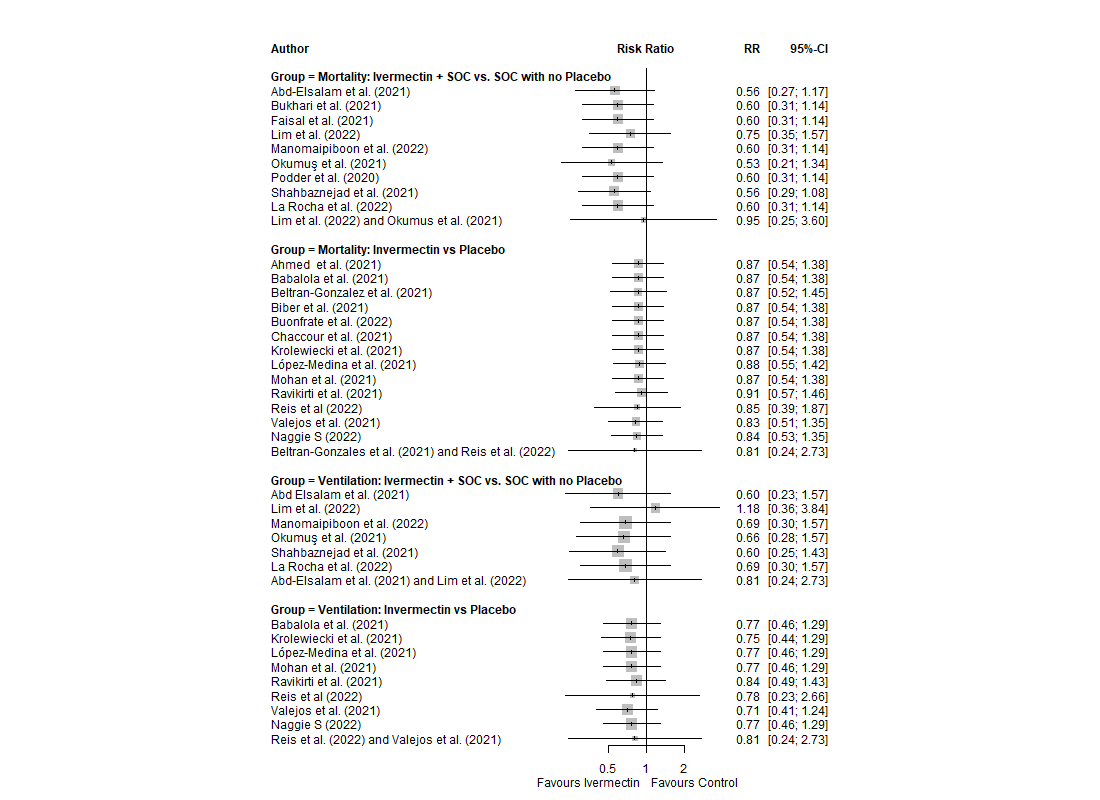

Supplement: Supplementary file 5 — Additional file 5. Forest plots of sensitivity analysis of mortality and invasive mechanical ventilation support. [file 12879_2022_7589_MOESM5_ESM.png]

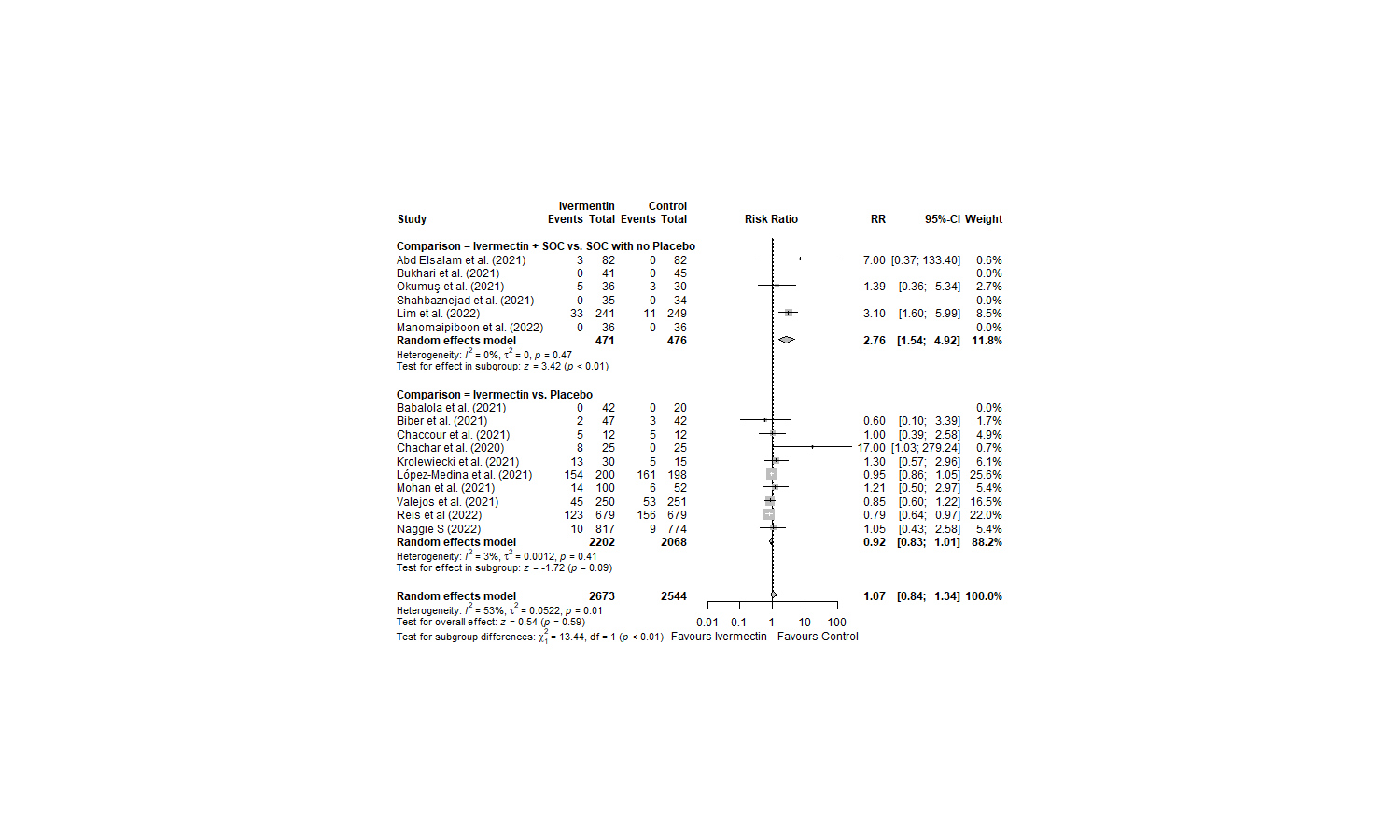

Supplement: Supplementary file 6 — Additional file 6. Forest plots showing the risk of adverse effects in patients who took ivermectin compared to controls, stratified by placebo or other drugs. RR: relative risk. [file 12879_2022_7589_MOESM6_ESM.png]
