# Supplementary material for: Systematic review and meta-analysis of ivermectin for treatment of COVID-19: evidence beyond the hype
Source: BMC Infect Dis. 2022 Jul 23;22:639. doi: 10.1186/s12879-022-07589-8 (PMC9308124; doi:10.1186/s12879-022-07589-8)
Supplement: Supplementary file 7 — Additional file 7. Search strategy. [file 12879_2022_7589_MOESM7_ESM.docx]

**Research Algorithms**

Following there are search strategies in different databases (PubMed/Medline, Embase, Cochrane Library and Lilacs)

***Search strategy for PubMed/Medline***

("Ivermectin"[MeSH Terms] OR "Stromectol"[All Fields] OR "Mectizan"[All Fields] OR "mk 933"[All Fields] OR "mk 933"[All Fields] OR "MK933"[All Fields] OR "Eqvalan"[All Fields] OR "Ivomec"[All Fields]) AND ("covid 19"[MeSH Terms] OR "covid 19"[All Fields] OR "covid 19 virus disease*"[All Fields] OR "COVID 19 Virus Disease"[All Fields] OR "disease covid 19 virus"[All Fields] OR "virus disease covid 19"[All Fields] OR "covid 19 virus infection"[All Fields] OR "covid 19 virus infection"[All Fields] OR "COVID-19 Virus Infections"[All Fields] OR "2019 ncov infection*"[All Fields] OR "2019 nCoV Infection"[All Fields] OR "infection 2019 ncov"[All Fields] OR "coronavirus disease 19"[All Fields] OR "coronavirus disease 19"[All Fields] OR "2019 Novel Coronavirus Disease"[All Fields] OR "2019 Novel Coronavirus Infection"[All Fields] OR "2019 ncov disease*"[All Fields] OR "2019 nCoV Disease"[All Fields] OR "disease 2019 ncov"[All Fields] OR "COVID19"[All Fields] OR "Coronavirus Disease 2019"[All Fields] OR "disease 2019 coronavirus"[All Fields] OR "SARS Coronavirus 2 Infection"[All Fields] OR "sars cov 2 infection"[All Fields] OR "infection sars cov 2"[All Fields] OR "sars cov 2 infection"[All Fields] OR "SARS-CoV-2 Infections"[All Fields] OR "covid 19 pandemic*"[All Fields] OR "COVID 19 Pandemic"[All Fields] OR "pandemic covid 19"[All Fields] OR "sars cov 2"[MeSH Terms] OR "Coronavirus Disease 2019 Virus"[All Fields] OR "2019 novel coronavirus*"[All Fields] OR "coronavirus 2019 novel"[All Fields] OR "novel coronavirus 2019"[All Fields] OR "Wuhan Seafood Market Pneumonia Virus"[All Fields] OR "sars cov 2 virus*"[All Fields] OR "SARS CoV 2 Virus"[All Fields] OR "virus sars cov 2"[All Fields] OR "2019-nCoV"[All Fields] OR "covid 19 virus*"[All Fields] OR "COVID 19 Virus"[All Fields] OR "virus covid 19"[All Fields] OR "Wuhan Coronavirus"[All Fields] OR "coronavirus wuhan"[All Fields] OR "SARS Coronavirus 2"[All Fields] OR "coronavirus 2 sars"[All Fields] OR "Severe Acute Respiratory Syndrome Coronavirus 2"[All Fields]) AND (("clinical"[Title/Abstract] AND "trial"[Title/Abstract]) OR "clinical trials as topic"[MeSH Terms] OR "clinical trial"[Publication Type] OR "random*"[Title/Abstract] OR "random allocation"[MeSH Terms] OR "therapeutic use"[MeSH Subheading])

***Search strategy for Embase***

#1 ‘ivermectin’/exp   OR (ivermectin/exp)

#2 ‘coronavirus disease 2019’/exp OR (coronavirus disease 2019/exp)

#3 'crossover procedure':de OR 'double-blind procedure':de OR 'randomized controlled trial':de OR 'single-blind procedure':de OR random*:de,ab,ti OR factorial*:de,ab,ti OR crossover*:de,ab,ti OR ((cross NEXT/1 over*):de,ab,ti) OR placebo*:de,ab,ti OR ((doubl* NEAR/1 blind*):de,ab,ti) OR ((singl* NEAR/1 blind*):de,ab,ti) OR assign*:de,ab,ti OR allocat*:de,ab,ti OR volunteer*:de,ab,ti

#4 #1 AND #2 AND #3

***Search strategy for Cochrane Library***

#1 MeSH descriptor: [Ivermectin] explode all trees 400 results

#2 MeSH descriptor: [COVID-19] explode all trees 337 results

# 3 #1 AND #2

***Search strategy for Biblioteca Virtual em Saúde (Lilacs, medRxiv, bioRxiv)***

#1  MH: "ivermectina" OR "ivermectin" OR "ivermectine"

#2  MH: "Infecções por Coronavirus" OR "Coronavirus Infections" OR "
Infecciones por Coronavirus" OR "COVID-19" OR "Doença pelo Novo Coronavírus (2019-nCoV)" OR "Doença por Coronavírus 2019-nCoV" OR "Doença por Novo Coronavírus (2019-nCoV)" OR "Epidemia de Pneumonia por Coronavirus de Wuhan" OR "Epidemia de Pneumonia por Coronavírus de Wuhan" OR "Epidemia de Pneumonia por Coronavírus de Wuhan de 2019-2020" OR "Epidemia de Pneumonia por Coronavírus em Wuhan" OR "Epidemia de Pneumonia por Coronavírus em Wuhan de 2019-2020" OR "Epidemia de Pneumonia por Novo Coronavírus de 2019-2020" OR "Epidemia pelo Coronavírus de Wuhan" OR "Epidemia pelo Coronavírus em Wuhan" OR "Epidemia pelo Novo Coronavírus (2019-nCoV)" OR "Epidemia pelo Novo Coronavírus 2019" OR "Epidemia por 2019-nCoV" OR "Epidemia por Coronavírus de Wuhan" OR "Epidemia por Coronavírus em Wuhan" OR "Epidemia por Novo Coronavírus (2019-nCoV)" OR "Epidemia por Novo Coronavírus 2019" OR "Febre de Pneumonia por Coronavírus de Wuhan" OR "Infecção pelo Coronavírus 2019-nCoV" OR "Infecção pelo Coronavírus de Wuhan" OR  "Infecção por Coronavirus 2019-nCoV" OR "Infecção por Coronavírus 2019-nCoV" OR "Infecção por Coronavírus de Wuhan" OR "Infecções por Coronavírus
Pneumonia do Mercado de Frutos do Mar de Wuhan" OR "Pneumonia no Mercado de Frutos do Mar de Wuhan" OR "Pneumonia por Coronavírus de Wuhan" OR "Pneumonia por Novo Coronavírus de 2019-2020" OR "Surto de Coronavírus de Wuhan" OR "Surto de Pneumonia da China 2019-2020" OR "Surto de Pneumonia na China 2019-2020" OR "Surto pelo Coronavírus 2019-nCoV" OR "Surto pelo Coronavírus de Wuhan" OR "Surto pelo Coronavírus de Wuhan de 2019-2020" OR "Surto pelo Novo Coronavírus (2019-nCoV) " OR "Surto pelo Novo Coronavírus 2019" OR "Surto por 2019-nCoV" OR
"Surto por Coronavírus 2019-nCoV" OR "Surto por Coronavírus de Wuhan" OR "Surto por Coronavírus de Wuhan de 2019-2020" OR  "Surto por Novo Coronavírus (2019-nCoV)" OR "Surto por Novo Coronavírus 2019" OR "Síndrome Respiratória do Oriente Médio" OR "Síndrome Respiratória do Oriente Médio (MERS)" OR "Síndrome Respiratória do Oriente Médio (MERS-CoV)" OR "Síndrome Respiratória do Oriente Médio por Coronavírus"

#3 #1 AND #2

***Search Strategy for the L.OVE platform***

coronavir* OR coronovirus* OR betacoronavir* OR "beta-coronavirus" OR "beta-coronaviruses" OR "corona virus" OR "virus corona" OR "corono virus" OR "virus corono" OR hcov* OR covid* OR "2019-ncov" OR cv19* OR "cv-19" OR "cv 19" OR "n-cov" OR ncov* OR (wuhan* AND (virus OR viruses OR viral)) OR "2019-ncov-related" OR "cv-19-related" OR "n-cov-related" OR sars* OR sari OR "severe acute respiratory syndrome" OR antisars* OR "anti-sars-cov-2" OR "anti-sars-cov2" OR "anti-sarscov-2" OR "anti-sarscov-2" OR "post-COVID-19" OR "Not-of-COVID-19" OR "corona patients" OR "article-covid-19" OR "post-covid-19" OR "post-covid" OR "with-covid-19" OR "pre-covid" OR "pre-covid-19" OR "with-covid" OR "anti-covid-19" OR "n-covid" OR "no-covid"

AND ivermectin*

Filter results by RCT

**Databases included in Living Overview of the Evidence (L-OVE) COVID-19 Repository by Epistemonikos Foundation**

MEDLINE; EMBASE; CINAHL; PsycINFO; LILACS (Latin American & Caribbean Health Sciences Literature); Wanfang Database; CBM - Chinese Biomedical Literature Database; CNKI - Chinese National Knowledge Infrastructure; VIP - Chinese Scientific Journal Database; IRIS (WHO Institutional Repository for Information Sharing); IRIS PAHO (PAHO Institutional Repository for Information Sharing)); IBECS - Índice Bibliográfico Español en Ciencias de la Salud (Spanish Bibliographic Index on Health Sciences); Microsoft Academic; ICTRP Search Portal; ClinicalTrials.gov; ISRCTN registry; Chinese Clinical Trial Registry; IRCT - Iranian Registry of Clinical Trials; EU Clinical Trials Register: Clinical trials for covid-19; NIPH Clinical Trials Search (Japan) - Japan Primary Registries Network (JPRN) (JapicCTI, JMACCT CTR, jRCT, UMIN CTR); UMIN-CTR - UMIN Clinical Trials Registry; JRCT - Japan Registry of Clinical Trials; JAPIC Clinical Trials Information; Clinical Research Information Service (CRiS), Republic of Korea; ANZCTR - Australian New Zealand Clinical Trials Registry; ReBec - Brazilian Clinical Trials Registry; CTRI - Clinical Trials Registry - India; RPCEC - Cuban Public Registry of Clinical Trials; DRKS - German Clinical Trials Register; LBCTR - Lebanese Clinical Trials Registry; TCTR - Thai Clinical Trials Registry; NTR - The Netherlands National Trial Register; PACTR - Pan African Clinical Trial Registry; REPEC - Peruvian Clinical Trial Registry; SLCTR - Sri Lanka Clinical Trials Registry; medRxiv; bioRxiv; SSRN Preprints; ChinaXiv; SciELO Preprints; Research Square.
